# Protective Effects of Chinese Traditional Medicine Longhu Rendan against Atherosclerosis via Negative Regulation of LOX-1

**DOI:** 10.1155/2018/4812639

**Published:** 2018-10-08

**Authors:** Sishan Yan, Teng Wu, Ning Li, Lingyi Zhang, Jun Song, Yunzheng Xu, Shumei Wang, Liqin Ding, Jiahua Jin, Ying Liu, Tian Lan

**Affiliations:** ^1^Guangdong Pharmaceutical University, Guangzhou, Guangdong 510006, China; ^2^Key Laboratory of Cardiovascular Disease and Department of Pathophysiology, Nanjing Medical University, Nanjing, Jiangsu 211166, China; ^3^Key Laboratory of Digital Quality Evaluation of Chinese Materia Medica of State Administration of TCM, Guangzhou 510006, China; ^4^Engineering & Technology Research Center for Chinese Materia Medica Quality of Guangdong Province, Guangzhou 510006, China; ^5^Shanghai Zhonghua Pharmaceutical Co., Ltd., Shanghai 201707, China

## Abstract

Longhu Rendan (LHRD), a Chinese traditional compound medicine, has a remarkable treatment effect on motion sickness for about half a century. However, the role of LHRD in atherosclerosis treatment is still unclear. In this study, LHRD treatment significantly diminished total cholesterol (TC), triglyceride (TG), and low-density lipoprotein cholesterol (LDL-C) levels in apolipoprotein E gene-knockout (ApoE^−/−^) mice fed with high fat and high cholesterol diet (western diet). Besides, LHRD treatment significantly reduced atherosclerotic lesion and plaques formation in both aortic roots and aortic trees. Furthermore, immunofluorescence staining in aortic roots demonstrated that LHRD treatment inhibited lectin-like oxidized low-density-lipoprotein receptor-1 (LOX-1) expression in atherosclerotic plaques. These results indicated that LHRD ameliorated atherosclerosis via reducing serum levels of TC, TG, and LDL-C as well as LOX-1 expression, subsequently attenuating atherosclerotic lesion and lipid deposition. In conclusion, LHRD could significantly attenuate experimental atherosclerosis and might be a novel potential drug for the prevention and treatment of atherosclerosis.

## 1. Background

Atherosclerosis, as a progressively chronic disease induced by complicated factors [1], accounts for the majority of morbidity and mortality of men and women in both developed and developing countries. It has been recognized that dyslipidemia participates in the initiation of atherosclerosis development [2]. Firstly, the dysfunction and structural alterations of endothelial cells (ECs) permit the accumulation of lipoproteins, which caused initial lipid deposition and an inflammatory response in the intima [3, 4]. Secondly, with the excessive lipid deposition in the intima, apoptotic or necrotic cell debris accumulate within the vessel wall of medium-sized and large arteries later [5–7]. Finally, dyslipidemia also promotes oxidative stress and inflammatory response such as cytokines and chemokines secret in plaques [8]. It has been reported that oxLDL internalization by macrophages through scavenging receptors promotes the progression of foam cell formation [9], suggesting that oxLDL may play a pivotal role in the development of atherosclerotic lesions [10, 11].

LOX-1, the dedicated receptor of oxLDL [12], belongs to E scavenger receptors, primary expressed on endothelial cells, smooth muscle cells, platelets, and macrophages [13–15]. LOX-1 has been considered as one of the major scavenger receptors in vascular endothelial cell [16] and mediated the recognition and internalization of oxLDL by vascular endothelial cells [17]. It has been recognized that LOX-1 takes part in the development of atherosclerosis, and inhibited LOX-1 expression could block the pathogenesis progression of atherosclerosis [18, 19].

As a Chinese traditional patent medicine, LHRD, has been used to relieve motion sickness for about a century. Recently, some studies have reported that* Glycyrrhiza* [20], as one of the major component of LHRD, could ameliorate the lipid metabolic dysfunction and exhibit anti-inflammatory activity through the regulation of ERK/NF-*κ*B/miR-155 signaling [21]. Other components, such as menthol [22] and* Eugenia caryophyllata* [23], also possess anti-inflammatory activity. However, few studies have investigated whether these components can regulate the progression of atherosclerosis. Herein, LHRD has been utilized for atherosclerosis treatment, and we hypothesized that LHRD could ameliorate atherosclerosis through downregulation of LOX-1 to inhibit lipid deposition in mice fed with high fat and high cholesterol diet.

## 2. Method

### 2.1. Materials and Reagents

LHRD was obtained from Shanghai Zhonghua pharmaceutical Co., Ltd. Atorvastatin (Ato) calcium, pills, was purchased from Pfizer Inc. Assay kits for TC, TG, and LDL-C were obtained from Jiancheng Institute of Biotechnology (Nanjing, China). Oil Red O was purchased from Sigma-Aldrich (St. Louis, USA). Hematoxylin and eosin reagents were purchased from Beijing Dingguo Changsheng Biotechnology Co. Ltd. LOX-1 antibody was from Abcam (Cambridge, UK).

### 2.2. Animal and Treatment

All animal procedures were conducted in accordance with the China Animal Welfare Legislation and were approved by the Ethics Committee on the Care and Use of Laboratory Animals in Guangdong Pharmaceutical University (Guangzhou, China). Eight 6-week-old male C57BL/6 mice (16-20 g) and 40 ApoE^−/−^ (16-20 g) mice were purchased from Experimental Animals Center of Guangdong Province, China. All ApoE^−/−^ mice were randomly divided into five groups: the atherosclerosis group, the positive control group (receiving 10 mg/kg/d Ato, i.g.), and LHRD treatment groups (receiving 100 mg/kg, 200 mg/kg, and 400 mg/kg LHRD, respectively, i.g.). All ApoE^−/−^ mice were induced to atherosclerosis with high fat and high cholesterol diet, and the C57BL/6 mice were fed with control diet. The high fat and high cholesterol diet, containing 19% protein, 18.5% fat, and 50.5% carbohydrate, were purchased from Experimental Animals Center of Guangdong Province, China. For the LHRD and the Ato groups, 0.5% sodium carboxyl methyl cellulose (CMC-Na) was used as vehicle (Veh) to dilute LHRD and Ato; meanwhile, the control and atherosclerotic mice received 0.5% CMC-Na i.g. in the ten-week period of the experiment.

### 2.3. Sample Preparations and Quantitation of Atherosclerosis

After ten weeks of experiment and with about 12 h fasting, all the mice were sacrificed to get the blood sample through the orbital vein. Then the mice were euthanized by cervical dislocation. All the tissues were flash frozen in liquid nitrogen and stored at -80°C for future analysis.

### 2.4. Biochemical Analysis

At the end of the experiment, animals were sacrificed for histological examination of the cardiac and artery. Blood was collected and centrifuged at 3000 rpm for 15 min, and then serum was separated to detect TC, TG, and LDL-C activities.

### 2.5. Quantitative Analysis of Atherosclerotic Lesions

Under integrated type microscope, the aortas were divided from the peripheric tissue and split to stain with Oil Red O. The hearts of the mice were embedded in optimal cutting temperature compound (OCT), OCT-embedded tissues were cut into 7 *μ*m sections by Leica freezing microtome when the root of aorta could be observed under microscope, and then tissues were stained with eosin (H&E) for routine histology and Oil Red O for lipid. The Oil Red O stain for both aorta and aortic root was performed using 0.3% Oil Red O (Sigma, St Louis, MO) working solution for 10 minutes and washed by 60% isopropanol for 30 seconds. The images of the aortas were captured (against black background) with a digital color camera while both the images of HE and Oil Red O of aortic root were captured by Olympus BX43 imaging. Percentage of Oil Red O-positive stained area in relation to total surface area was quantified using computer-assisted morphometry Image-Pro Plus software (IPP6.0).

### 2.6. Immunofluorescent Staining

Immunofluorescence (IF) staining for LOX-1 in aortic root was performed using 1% bovine serum albumin (BSA) to cover the false positive area, afterwards washed by PBS, and primary antibodies were incubated overnight at 4°C. The next day, samples were incubated with a green fluorescence-labeled polymer conjugated to secondary antibodies. After staining by DAPI dihydrochloride for 10 minutes, the mounting medium was used to seal the tissue section and the images were captured by Leica DMi8 imaging. Percentage of immunofluorescence-positive stained area in relation to total surface area was quantified using computer-assisted morphometry ImageJ.

### 2.7. Statistical Analysis

Statistical significance of differences was calculated using one-way ANOVA with Bonferroni post hoc for multiple-group comparison or unpaired. The analyses were performed using GraphPad Prism 6.0 software;* P*<0.05 was considered to be statistically significant.

## 3. Results

### 3.1. Effect of LHRD on Body Weight and Metabolic and Biochemical Parameters in ApoE^−/−^ Mice

**Figure 1:**
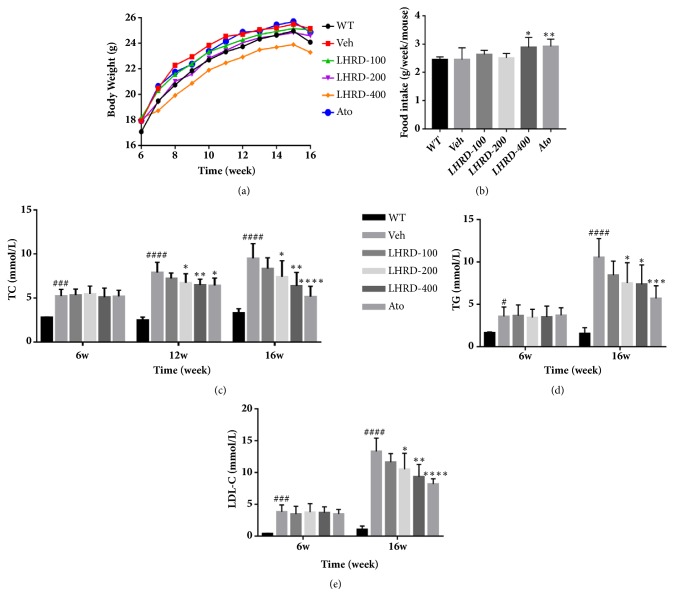
LHRD improves metabolic parameters of ApoE^−/−^ mice. All ApoE^−/−^ mice were fed with high fat and high cholesterol diet for ten weeks. Meanwhile, all ApoE^−/−^ mice at 6 weeks were intragastrically administered LHRD (100, 200, 400 mg/kg) or vehicle for 10 weeks, and age-matched C57BL/6J mice were used as control. ((a) and (b)) Average body weight and food intake of each group. ((c), (d), and (e)) Serum levels of TC, TG, and LDL-C. Data are means ± SD, ^#^*P*<0.05, ^###^*P*<0.001, and ^####^*P*<0.0001 compared to WT group (n=8 per group). ^∗^*P*<0.05, ^∗∗^*P*<0.01, ^∗∗∗^*P*<0.001, and ^∗∗∗∗^*P*<0.0001 compared to Veh group (treated with vehicle control). Ato: atorvastatin, LHRD: Longhu Rendan.

As a well-known atherosclerotic animal model, ApoE^−/−^ mice developed foam cell-rich depositions in their aortas by the age of 3 months and ultimately developed atherosclerotic lesions similar to human atherosclerosis [24]. In this study, ApoE^−/−^ mice were fed with western diet for 10 weeks to evaluate the effect of LHRD on body weight and serum lipid metabolism. As shown in Figures 1(a) and 1(b), the average body weight of the group for 400 mg/kg LHRD treatment is lower than the model group, although the food intake was increased contrarily. In addition, the further studies for serum biochemical analysis proved that the serum levels of TC, TG, and LDL-C of ApoE^−/−^ mice were remarkably (*P*<0.0001) higher than wild type mice after fed with western diet (Figures 1(c)–1(e)). Meanwhile, LHRD, especially the dosage of 400 mg/kg group (*P*<0.01), and the serum TC, TG, and LDL-C of ApoE^−/−^ mice have been decreased by about 30% compared to the model group, although the effect of series of dosages of LHRD was not as potential as Ato (*P*<0.0001).

### 3.2. LHRD Attenuates the Lesion Area of Aorta in ApoE^−/−^ Mice

**Figure 2:**
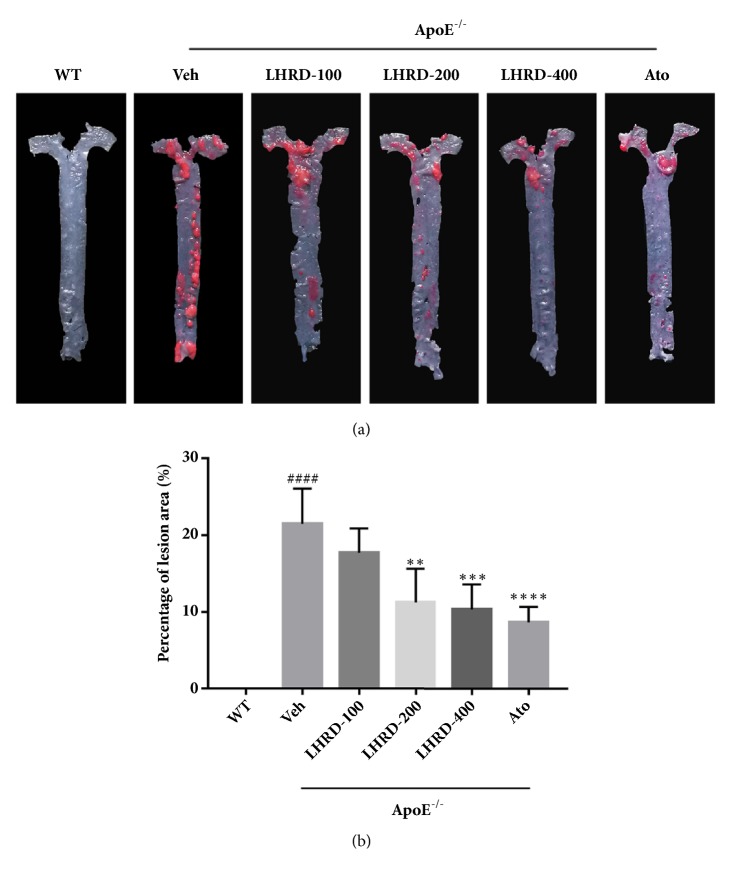
LHRD reduces the lesion area in aorta. (a) Representative histology of Oil Red O staining in aorta. (b) Quantification of positive staining areas was measured by Image-Pro Plus 6.0 software. Data are means ± SD, ^####^*P*<0.0001 compared to WT group (n=8 per group). ^∗∗^*P*<0.01, ^∗∗∗^*P*<0.001 and ^∗∗∗∗^*P*<0.0001 compared to Veh group (treated with vehicle control). Ato: atorvastatin, LHRD: Longhu Rendan.

Subendothelial accumulations of cholesterol-engorged macrophages and the foam cell formation have one of the most pivotal roles in the progression of atherosclerosis [25]. To examine the role of LHRD in atherosclerosis, Oil Red O staining for aortic trees was used to evaluate the atherosclerotic lesions. As shown in Figure 2, LHRD treatment (400 mg/kg/d) can reduce atherosclerotic lesions in the aorta up to 50% compared with ApoE^−/−^ mice treated with vehicle manifested atherosclerotic lesions, which indicated the antiatherosclerotic plaques lesions effect of LHRD.

### 3.3. LHRD Reduces the Lesion Area of Aortic Root in ApoE^−/−^ Mice

**Figure 3:**
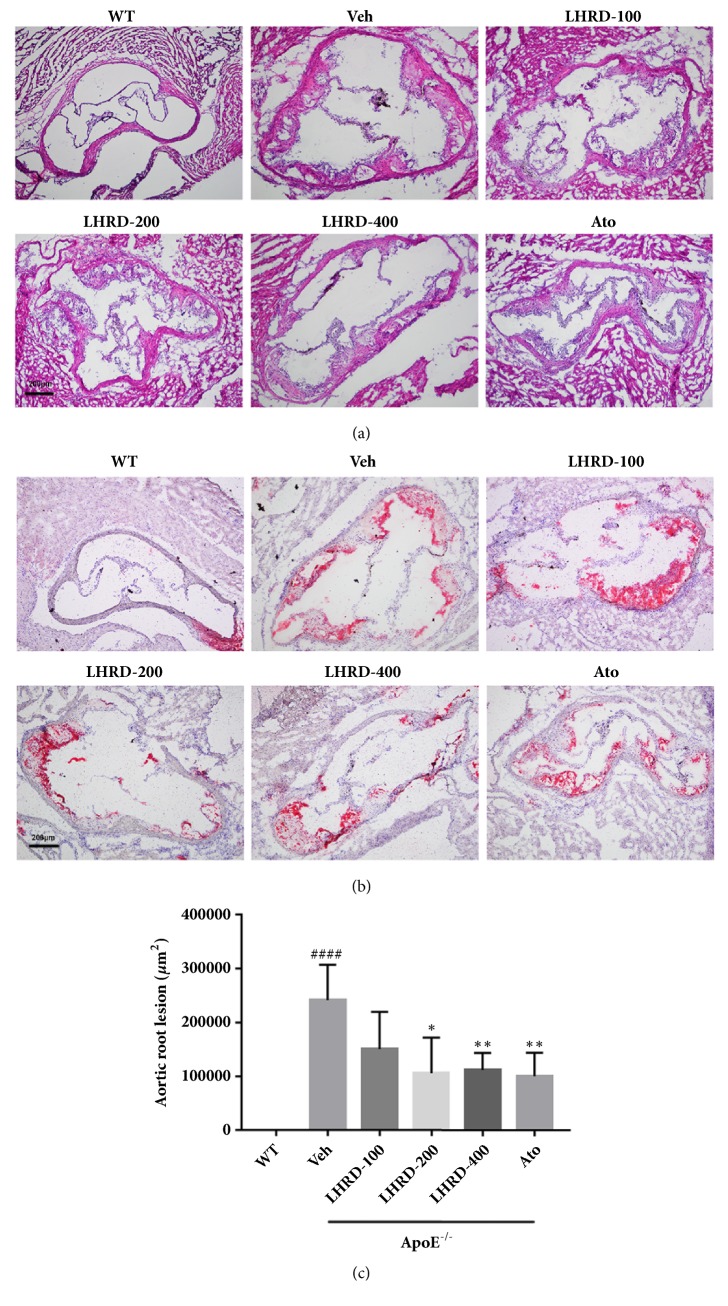
LHRD reduces the lesion area in aortic root. ((a) and (b)) Representative histology of H&E and Oil Red O staining in aortic root. Original magnification X10. (c) Quantification of positive staining areas was measured by Image-Pro Plus 6.0 software. Data are means ± SD, ^####^*P*<0.0001 compared to WT group (n=8 per group). ^∗^*P*<0.05 and ^∗∗^*P*<0.01 compared to Veh group (treated with vehicle control). Ato: atorvastatin, LHRD: Longhu Rendan.

In order to further examine the therapeutic effect of LHRD on atherosclerotic plaques in aortic root, we next performed HE and Oil Red O staining. HE staining results (Figure 3(a)) showed that LHRD treatment markedly decreased lipid deposition in the aortic root of ApoE^−/−^ mice. Furthermore, Oil Red O staining in Figure 3(b) proved that LHRD treatment (400 mg/kg/d) significantly (*P*<0.01) diminished plaque formation in the aortic root of ApoE^−/−^ mice with the dose dependent effect.

### 3.4. LHRD Diminishes LOX-1 Expression in Aortic Root of ApoE^−/−^ Mice

**Figure 4:**
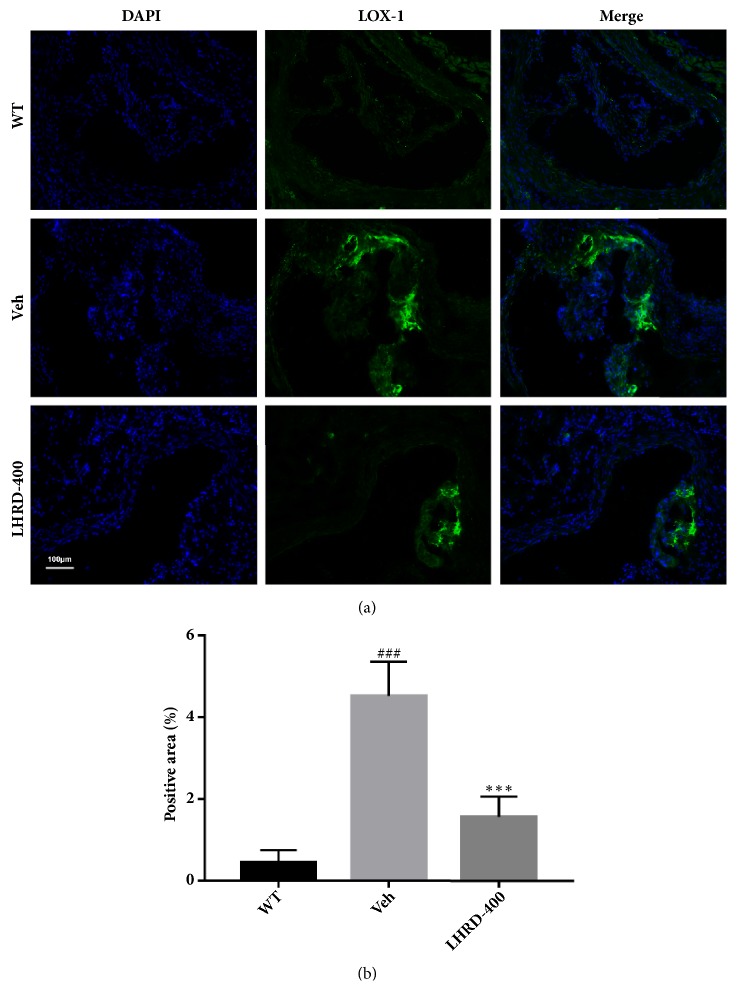
LHRD downregulates the protein expressions of LOX-1 in aortic root. (a) Representative histology of immunofluorescence staining of LOX-1 in aortic root. Original magnification X20. (b) Quantification of positive staining areas was measured by Image J software. Data are means ± SD, ^###^*P*<0.001 compared to WT group (n=8 per group). ^∗∗∗^*P*<0.001 compared to Veh group (treated with vehicle control). Ato: atorvastatin, LHRD: Longhu Rendan.

LOX-1, a scavenger receptor mediating the recognition and internalization of oxLDL in vascular endothelial cells, has been considered to participate in the progression of atherosclerosis via lipid metabolic regulation. To verify the underlying mechanism of the therapeutic effects on LHRD treatment, immunofluorescence was used to detect the expression of LOX-1 in aortic root. As shown in Figure 4, the expression of LOX-1 in the aortic root was dramatically (*P*<0.001) attenuated by LHRD treatment (400 mg/kg/d) compared with vehicle treatment control in ApoE^−/−^ mice, indicating that LHRD ameliorates atherosclerosis through downregulation of LOX-1.

## 4. Discussion

Our current study demonstrates that LHRD ameliorates biochemical metabolism, lipid deposition, and arterial injury in western diet-fed ApoE^−/−^ mice through downregulation of LOX-1.

ApoE^−/−^ mouse is created by homologous recombination in Embryonic stem cells (ES cells) [26]. Being knockout of the apolipoprotein E gene [27], it can easily develop atherosclerotic lesions in the aorta and coronary and pulmonary arteries under the stimulation of western diet [28]. As a traditional compound medicine, LHRD has been used to relieve motion sickness for over half a century. In this subject, it has been demonstrated that LHRD ameliorated atherosclerosis through inhibition of atherosclerotic lesions and plaque formation both in aortic roots and aortic trees of ApoE^−/−^ mice fed with western diet. Especially, high dose of LHRD had similarly beneficial effects on ApoE^−/−^ mice as what atorvastatin did, which proved the antiatherosclerosis effect of LHRD.

As important components of LHRD, menthol and the root of Aucklandia lappa Decne [29] exert anti-inflammatory activity through reducing inflammatory cytokines such as TNF-*α* while* Eugenia caryophyllata* exhibits its function through its regulation in adhesion molecules. Besides, it is reported that Fructus Amomi could significantly inhibit heart inflammation [30]. Due to the significant biological properties of component of LHRD, we speculate that LHRD can reduce LOX-1 through the anti-inflammation activity of its component and exert its function against atherosclerosis. In this study, we evaluated the antiatherosclerotic effects of LHRD in mice fed with western diet for 10 weeks. LHRD treatment significantly reduced plaques lesions in aortic roots and aortic trees.

Atherosclerosis, the main etiological factor of coronary heart disease, cerebral infarction, and peripheral vascular diseases, is a continuously chronic metabolism disease characterized by the deposition of excessive cholesterol in the arterial intima [31]. It has been reported that lipid metabolism disorder began in the intima and generally makes accumulation of lipid and compound carbohydrate, which finally promote the initiation and development of atherosclerosis [32–34]. Triglycerides are the most abundant lipids in the body and increasing evidence has shown that hypertriglyceridemia is associated with AS [35]. Excess cholesterol makes macrophages turn into “foam cells” [36], so it is regarded as the root cause of atherosclerosis. Besides, increased LDL-C is an independent risk factor for atherosclerosis and reducing of LDL-C is the most basic therapeutic target for atherosclerosis. Hence, regulation of serum lipid is the key to treating atherosclerosis. In the current study, it has been demonstrated that LHRD protects against atherosclerosis via attenuation of serum TC, TG, and LDL-C levels, suggesting the ameliorating lipid disorder effect of LHRD. Statins can stabilize vulnerable atherosclerotic plaques and plays a critical role in exerting anti-inflammatory and atheroprotective effects. However, the side effects associated with stains, such as predispose to incident cataract in the general population, limit their usage [37]. Our current study showed the similar therapy effect of 400 mg/kg LHRD as the atorvastatin. As a traditional Chinese medicine, the better security of LHRD allows it being used in daily therapy. The combination therapy of LHRD and stains can be research in the future and may provide a new direction of atherosclerosis therapy.

Atherosclerosis is a complex pathophysiological process and the formation of foam cells is a key step in the development and progression of atherosclerosis. As series of cell surface receptors, scavenger receptors are thought to participate in the progression of atherosclerosis via mediated lipid internalization into cells. As one of the primary scavenger receptors for the initiation and progression of atherosclerosis, LOX-1 has been identified as a major receptor for oxLDL in endothelial cells, monocytes, platelets, cardiomyocytes, and vascular smooth muscle cells [38, 39]. In endothelial cells, oxLDL-LOX-1 interaction causes endothelial dysfunction through increasing the expression of cell adhesion and activating apoptotic pathways [40–42]. In a proinflammatory environment, rising LOX-1 contributes to more oxLDL uptake and promotes lipid accumulation, and significant rise in ROS in macrophages leads to inhibiting macrophage migration and foam cell formation [43]. The upregulation of LOX-1 levels can activate the transcription factor NF-*κ*B, which stimulates inflammatory cytokines (TNF-*α*, IL-6, IL-I *β*, etc.) release in plaque lesions. Further studies also indicated that LOX-1 upregulation in endothelial cells promotes cell adhesion molecules (VCAM-1, etc.), monocyte chemoattractant protein (MCP-1), and inflammation related proteins expression, which advanced the development of atherosclerosis. Therefore, substances and drugs with anti-inflammatory activities would prevent the formation of atherosclerosis and own the function of cardiovascular protection. In this subject, reduction of LOX-1 expression in aortic plaques lesions of ApoE^−/−^ mice treated by LHRD is obviously significant, suggesting that LHRD attenuates atherosclerosis through downregulation of LOX-1.

## 5. Conclusion

LHRD treatment significantly decreased the atherosclerotic lesions via ameliorated serum lipid disorder and LOX-1 expression in plaques, indicating that LHRD may represent a novel therapeutic approach for preventing the progression of atherosclerosis.

## Data Availability

The data used to support the findings of this study are available from the corresponding author upon request.
